# The reporting checklist for public versions of guidelines: RIGHT-PVG

**DOI:** 10.1186/s13012-020-01066-z

**Published:** 2021-01-11

**Authors:** Xiaoqin Wang, Yaolong Chen, Elie A. Akl, Ružica Tokalić, Ana Marušić, Amir Qaseem, Yngve Falck-Ytter, Myeong Soo Lee, Madelin Siedler, Sarah L. Barber, Mingming Zhang, Edwin S. Y. Chan, Janne Estill, Joey S. W. Kwong, Akiko Okumura, Qi Zhou, Kehu Yang, Susan L. Norris, The RIGHT working group

**Affiliations:** 1 Michael G. DeGroote Institute for Pain Research and Care, McMaster University, Hamilton, ON L8S 4 K1 Canada; 2 Evidence-Based Medicine Center, School of Basic Medical Sciences, Lanzhou University, Lanzhou, 730000 China; 3 WHO Collaborating Center for Guideline Implementation and Knowledge Translation, Lanzhou, 730000 China; 4 Department of Internal Medicine, American University of Beirut, Beirut, Lebanon; 5 Department of Research in Biomedicine and Health, University of Split School of Medicine, Split, Croatia; 6 American College of Physicians, Philadelphia, PA USA; 7 Division of Internal Medicine, Louis Stokes Veterans Affairs Medical Center, Case Western Reserve University, Cleveland, OH USA; 8 Clinical Medicine Division, Korea Institute of Oriental Medicine, Daejeon, 34054 Republic of Korea; 9 Division of Physical Education and Exercise Science, University of South Florida, Tampa, FL USA; 10 World Health Organization Centre for Health Development, Kobe, 651-0073 Japan; 11 Chinese Evidence-Based Medicine Centre, West China Hospital, Sichuan University, Chengdu, 610041 China; 12 Cochrane Singapore, Singapore Clinical Research Institute, Consortium for Clinical Research & Innovation Singapore, Singapore, Singapore; 13 Centre for Quantitative Medicine, Duke-NUS Medical School, Singapore, Singapore; 14 Institute of Global Health, University of Geneva, Geneva, Switzerland; 15 Mathematical Statistics and Actuarial Science, University of Bern, Bern, Switzerland; 16 United Nations Population Fund Asia and the Pacific Regional Office, Bangkok, Thailand; 17 Medical Information Network Distribution Service (MINDS) Guideline Centre, Tokyo, Japan; 18 Oregon Health & Science University, Portland, OR USA

**Keywords:** Guideline, Public or patient version of guidelines (PVG), Reporting quality, Reporting checklist

## Abstract

**Background:**

Public or patient versions of guidelines (PVGs) are derivative documents that “translate” recommendations and their rationale from clinical guidelines for health professionals into a more easily understandable and usable format for patients and the public. PVGs from different groups and organizations vary considerably in terms of quality of their reporting. In order to address this issue, we aimed to develop a reporting checklist for developers of PVGs and other potential users.

**Methods:**

First, we collected a list of potential items through reviewing a sample of PVGs, existing guidance for developing and reporting PVGs or other similar evidence-based patient tools, as well as qualitative studies on original studies of patients’ needs about the content and/or reporting of information in PVGs or similar evidence-based patient tools. Second, we conducted a two-round Delphi consultation to determine the level of consensus on the items to be included in the final reporting checklist. Third, we invited two external reviewers to provide comments on the checklist.

**Results:**

We generated the initial list of 45 reporting items based on a review of a sample of 30 PVGs, four PVG guidance documents, and 46 relevant studies. After the two-round Delphi consultation, we formed a checklist of 17 items grouped under 12 topics for reporting PVGs.

**Conclusion:**

The RIGHT-PVG reporting checklist provides an international consensus on the important criteria for reporting PVGs.

**Supplementary Information:**

The online version contains supplementary material available at 10.1186/s13012-020-01066-z.

Contributions to the literature

- Our research is the first literature and consensus-based reporting checklist for public or patient versions of guidelines.
- The RIGHT for PVG reporting checklist can help to improve the transparency, consistency, and usability of their guidelines, which may ultimately lead to better uptake of clinical guidelines, improved shared decision-making between patients and clinicians, and improved patient health outcomes.

## Introduction

The availability of trustworthy health-related information for patients can promote informed and shared decision-making and improve health outcomes [1]. Clinical guidelines represent a reliable source of information for health professionals. However, guidelines usually use very technical language, making it challenging for patients and the public to understand and use, and therefore, researchers have suggested that patient-friendly guidelines products should be developed [2]. Patient versions of guidelines (PVGs) are “documents that ‘translate’ guideline recommendations and their rationales originally produced for health professionals into a form that is more easily understood and used by patients and the public” [3]. PVGs should provide reliable, concise, and easy to understand information for patients. In addition, PVG may be helpful to make people more reassured and confident about their care [3].

International guideline organizations, such as the American College of Physicians (ACP), the National Institute for Health and Care Excellence (NICE), the Scottish Intercollegiate Guidelines Network (SIGN), the American Academy of Neurology (AAN), the National Comprehensive Cancer Network (NCCN), and the US Preventive Services Task Force, are aware of the importance of PVGs and have developed their own PVGs [4–8]. However, the content reported in PVGs from different organizations varies significantly [9]. Moreover, we could not identify any systematically developed checklists for PVG reporting.

Reporting checklists can promote transparent and rigorous reporting of guidelines [10]. The Reporting Tool for Practice Guidelines in Health Care (RIGHT) is a checklist for clinical guidelines [11]. As PVGs differ from guidelines with respect to the target audience, aims, scope, wording, and reporting style, they require a dedicated checklist [3, 9]. Formal, carefully constructed reporting checklists can improve the quality of reporting of PVGs and promote their use in enhancing communication between patients and healthcare practitioners.

## Methods and analysis

### Objective

The aim of our study was to identify essential reporting items for PVGs to promote transparency, and to optimize their use and ultimately their impact on outcomes that matter to patients.

### Development process

The detailed methods used to develop the RIGHT for PVG have been reported previously [12]. For the overall development process, the following stages were applied.

### Collecting initial items

We generated the initial list of items through systematically reviewing (1) a sample of PVGs, (2) existing guidance for conducting and reporting PVGs or similar evidence-based patient tools, and (3) original studies of patients’ needs about the content and/or reporting information of PVG or similar evidence-based patient tools, which could contribute to the checklist from the perspective of patients and the public. The detailed inclusion criteria for articles of interest can be found in the published protocol [12].

We took a sample of one to two PVGs from each organization that has developed PVGs [12]. We included PVGs that were (1) defined as patient or public versions by the developers and (2) freely available to the public. This led to a sample of 30 PVGs for reviewing (see Additional file 1: Appendix 1).

To identify existing guidance for conducting and reporting PVGs, we searched the guidance documents for PVGs on the official websites of organizations that had published PVGs. When such a document was not available, we attempted to contact the person(s) named on the website.

To identify studies on patients’ needs and studies relevant to the reporting and conducting of PVGs, we searched PubMed without language restrictions. We developed the search strategies with the assistance of an information scientist (Junqiao Chen, University of Oxford) (see Additional file 1: Appendix 2). Two researchers (XW and QZ) independently screened the titles and abstracts for all potentially eligible studies, and then reviewed the full text against the eligibility criteria. Given that diverse names were used to describe PVGs, we also searched the reference lists and citations and used the “Similar Articles” function in PubMed as a form of snowball search [4]. We also contacted experts in relevant fields and used the Google search engine to supplement the search.

### Delphi consultation

Seventeen experts with technical expertise in guideline development, PVGs, GRADE (Grading of Recommendations Assessment, Development and Evaluation), plain language editing, and reporting guidelines, epidemiology as well as physicians and three representatives of the public with previous experience in guideline-related work agreed to participate in the Delphi consultation (Additional file 1: Appendix 3). Our experts were from the USA, China, Croatia, Japan, Korea, Lebanon, Norway, Singapore, and Switzerland.

To achieve consensus on which items to include, two rounds of a modified Delphi consultation were conducted [13, 14]. The panelists used a 7-point Likert scale to express their agreement with including each item. During the Delphi consultation, an item was included when 75% or more of participants chose 6 or 7 on the Likert scale. When less than 75% of the Delphi participants selected a score of 6 or 7, we admitted items into the next round if 80% or more of panelists chose scores from 4 to 7 and excluded the item if this level of agreement was not achieved. In addition, we excluded items when 75% or more of participants rated the item 1 to 3. The panelists were asked to suggest any additional items they thought potentially relevant in the first round. The second round of the survey included items with no consensus and any new items proposed by at least one respondent in the first round. During both rounds, we modified some items considering comments from the panelists and provided the panelists with a summary of results after each round.

One researcher (YY) analyzed the results of the Delphi consultation without having access to the name of the Delphi panelist. We had several discussions about how to present the checklist, after which all the panelists were invited to review the checklist for further comments.

### External review

We invited two external reviewers (JC and HSA) with relevant expertise in clinical guideline methods and knowledge translation of guidelines to review the checklist. Their comments are reflected in the final checklist.

## Results

### Generation of the RIGHT for PVG checklist

We generated the initial topics and relevant items from a sample of 30 PVGs. Then, we identified four PVG guidance documents and summaries from organizations developing PVGs (Additional file 1: Appendix 4) and used them to refine the topics. A total of 34 overarching topics were thus generated.

Out of 3680 records captured in PubMed, 211 articles met the eligibility criteria after title and abstract screening. After reading the full texts, we included 19 articles. The review of the reference lists of these articles revealed an additional 45 studies. Eighteen studies were excluded because no items could be summarized from them; thus, 46 studies were included from which 45 initial items were generated under the 34 topics (Additional file 1: Appendix 5).

We circulated a checklist of the 45 initial items to the panelists in the first round of the Delphi consultation. Of these, 39 items were about the content that needed to be reported in PVGs, and six focused on other aspects, including publication medium, modality, and presentation style of a PVG. In the first round of the Delphi consultation, 16 panelists responded and 14 items about the content of PVGs were included. As the desire was to focus on the content of PVGs, we excluded items related to publication medium (e.g., paper or web page), modality (e.g., text or figures), and style (e.g., presentation of text or numbers). We excluded some items related to format; however, the Delphi panel prioritized one item related to format (i.e., highlighting the recommendations), which was deemed to have a substantial impact on the use of the guideline. Different organizations often have a unique style for their products and PVGs also need to consider the special characteristics of their target patients or public groups when choosing an optimal style, so the format and style can be expected to vary. In the second round, 17 panelists responded and four items were included. Thus, a total of 18 items were included after Delphi consultation.

**Fig. 1:**
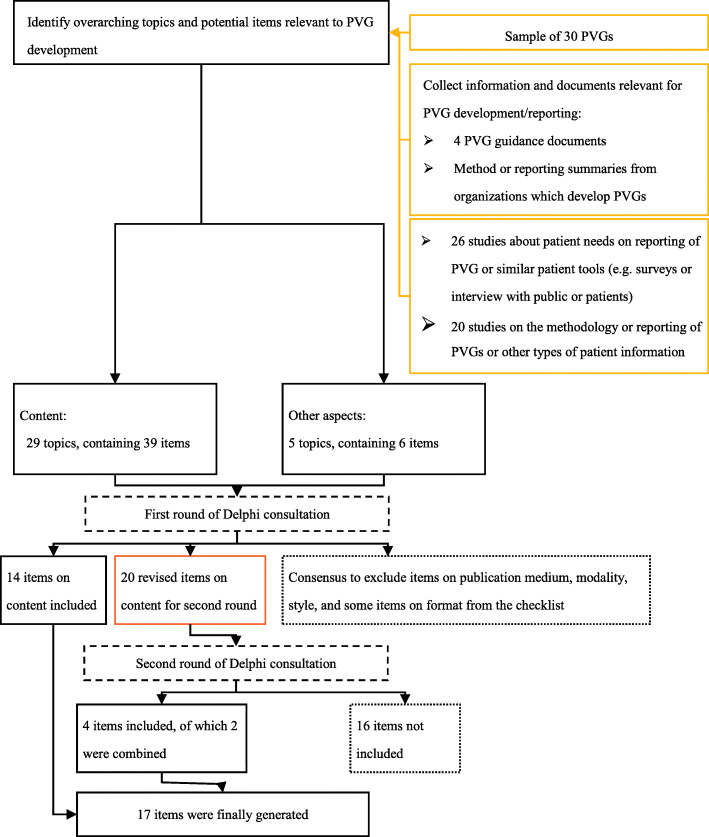
Flowchart of the item generation

Based on the results of the Delphi consultation and further discussions, we finalized the checklist. Two items on the same topic were combined into one. All panelists who participated in the Delphi consultation reviewed and approved the final checklist of 17 items. The overall generation process of item selection is shown in Fig. 1.

We added one or two examples for each item and sent them for review and confirmation by the panelists in the Delphi group. Meanwhile, the two invited external reviewers commented on our draft including the checklist, resulting in further refinements. The final checklist together with examples is shown in Table 1. Items where consensus could not be reached are listed in Additional file 1: Appendix 6.

**Table 1:** RIGHT for PVG checklist

Section/topic	RIGHT for PVG checklist
**Basic information**	
**1. Title/cover/copyright**	1.1 Identify the document as a guideline version for patients and the public. **Example** Health services for people with sarcoma: Understanding NICE guidance—information for the public [15] Parent Information Queensland Clinical Guidelines: Breastfeeding your baby [16]
	1.2 Specify the topic (e.g., condition, technique, or medication) addressed in the PVG. **Example** Health services for people with sarcoma: Understanding NICE guidance—information for the public [15]
	1.3 Specify the publication year and the version (if applicable) (e.g., first version, second version) of the PVG in the title, cover page, or copyright statement. Examples Kidney Cancer-NCCN Guidelines for Patients® Version 1.2015 [17]
**2. Contact information**	2.1 Provide contact information of the developers of the PVG (e.g., affiliations, website, or address, phone number, or email address). **Example** You can read more about us by visiting www.sign.ac.uk or you can phone 0131 623 4720 and ask for a copy of our booklet “SIGN guidelines: information for patients, carers and the public.” [18]
**3. Summary**	3.1 Provide a summary of the PVG, including the main recommendations. **Example** [19] Key points • Breast cancer is rare in pregnancy. • Most women who become pregnant after treatment for breast cancer have healthy pregnancies and healthy babies. • If you have breast cancer, you will be looked after by a specialist team who will discuss your treatment options with you. • If you are diagnosed with breast cancer while you are pregnant, your treatment will usually begin straight away. Neither the medications used nor surgery will harm your baby. You may have further treatment after your baby is born. • If you hope to have a baby in the future, your treatment plan can take your wishes into account. • It is usually safe to breastfeed after breast cancer, although surgery and radiotherapy may make it difficult. • If you have had treatment for breast cancer, you may be advised to wait for 2 years before becoming pregnant.
**Background**	
**4. Introduction of the target topic**	4.1 Introduce the target condition, including (as relevant) the definition, risk factors, signs, subtypes, complications, staging (progress), and epidemiology. **Example** [17] *Part 1* Kidney cancer basics. What are the kidneys? How does kidney cancer start? How does kidney cancer spread? Review
	4.2 Introduce the management, preventive, diagnostic, and other options. **Examples** 1) Why is breastfeeding important? [16] 2) What are behavioral and pharmacotherapy interventions? [20]
**5. Purpose, scope, and target users**	5.1 Describe the scope, purpose, intended use, and users of the PVG. **Example** [17] How to use this book: Who should read this book? This book is about treatment for renal cell carcinoma—the most common type of kidney cancer in adults. It does not discuss transitional cell carcinoma, Wilms tumor, or renal sarcoma. Patients and those who support them—caregivers, family, and friends—may find this book helpful. The information in this book may help you talk with your treatment team, understand what doctors say, and prepare for treatment Does the whole book apply to me? The recommendations in this book are based on science and the experience of NCCN experts. However, each patient is unique and these specific recommendations may not be right for you. Your doctors may suggest other tests or treatments based on your health and other factors. This book does not replace the knowledge and suggestions of your doctors.
**6. Link to the source guideline**	6.1 Provide a reference or link to the source guideline of the PVG, where the methods of the source guideline (e.g., the evidence review and recommendation development process) can be found. **Examples** 1) If you would like to see the clinical guideline, please visit www.sign.ac.uk [18]. 2) The medical information described in this document is based on the clinical practice guidelines of the European Society for Medical Oncology (ESMO) for the management of stomach cancer. It has been written by a medical doctor and reviewed by two oncologists from ESMO including the lead author of the clinical practice guidelines for professionals. It has also been reviewed by patients’ representatives from ESMO’s Cancer Patient Working Group [21].
**Recommendations**	
**7. Recommendations**	7.1 Include for each recommendation: (a) the target populations or conditions; (b) the recommended treatment or management option (e.g., prevention plan, diagnostic strategy, or rehabilitation); (c) potential benefits and harms, especially those that are patients important; and (d) the specific settings where the options are recommended to be implemented **Examples** *1) What can I do to help myself?* How often should I have my eyes checked? [18] If eye tests have shown that you have increased eye pressure, you should have your eyes checked every 2 years to make sure there is no glaucoma (strong recommendation) If you have a close relative (e.g., brother, sister, mother or father) who has glaucoma, you should have a review every 2 years. If you also have other risk factors (outlined on page 8), you should have your eyes checked for signs of glaucoma every year (recommendation). Should I have a patient-held record? There is not enough research evidence to tell us if a patient-held record (a patient’s personal copy of their glaucoma medical records) is of benefit to patients who have or are at risk of glaucoma. Some people may find having one helpful, but other people may not (not enough research evidence to tell us if something is of benefit). *2) Key recommendation*s: Prompt referral for expert diagnosis is crucial [15]
	7.2 Describe what options, if any, are available to deal with undesirable outcomes. **Example** [21] *What happens after treatment?* It is not unusual to experience treatment-related symptoms once the treatment is over. ● It is not rare that anxiety, sleeping problems, or depression are experienced in the post-treatment phase. Patients who experience these symptoms may benefit from psychological support. ● Memory deficiencies and difficulties in concentrating are common side effects of chemotherapy* and are generally reversible within a few months. ● Fatigue can last for months after treatment. Most patients find their energy levels are back to normal within 6 months to a year. After gastrectomy, the patient has to develop new eating habits. A nutritionist* can help patients adjust to this. Due to the removal of the upper part of the stomach, the body will absorb less vitamin B12 from food. Regular blood tests are advised, and often substitution with vitamin B12 injections is necessary. It is common to have diarrhea for some months after stomach surgery. Some patients also suffer from heartburn and abdominal pain. Removal of the spleen may lead to a reduced immunity. Therefore, the patient will receive several vaccinations, before and after the removal of the spleen and antibiotics to take every day. It is also important to be aware that any infection carries a greater risk and should be a reason to see a doctor and sometimes start taking antibiotics.
	7.3 Describe the self-management options, if any are reported in the source guideline. **Example** [18] *What can I do to help myself?* Have your eyes tested regularly Glaucoma is often picked up by a routine eye test so you should have your eyes checked regularly. When you have an eye test, your optometrist will check your sight and will look for signs of eye disease such as glaucoma. You should have the routine tests described on page 13. The cost of an eye test is covered by the NHS so it is FREE when you have it.
**8. The strength of the recommendations and certainty of the evidence**	8.1 Provide a clear and simple explanation of the meaning of terms related to the strength of recommendations and quality of the evidence (e.g., by using commonly understood symbols). **Examples** [18, 20] 1) Use to express strong recommendation, use to represent recommendation, and use to say not enough evidence (see page 3 in reference [18]). 2) Use Grade A, B, C, D, I statement to stand for recommended, recommended, recommendation depends on the patient’s situation, not recommended, and not enough evidence to make a recommendation (see page 4 in reference [20]).
**Other information**	
**9. Questions to ask**	9.1 Suggest a list of questions for patients to ask their healthcare providers if relevant. **Example** [17] Questions to ask your doctors Questions about testing 1) What tests will I have? How often will I be tested? 2) Where will the tests take place? Will I have to go to the hospital? 3) How long will it take? Will I be awake? 4) Will it hurt? Will I need anesthesia? 5) What are the risks? What are the chances of infection or bleeding afterward? 6) How do I prepare for testing? Should I not take aspirin? Should I not eat beforehand?
**10. Terms and abbreviations**	10.1 Provide a list of terms and abbreviations used in the PVG. **Example** [22] Part 11: Dictionary **Ablation**: Removal of diseased or unwanted tissue by surgery or other means. **Add-back therapy**: Hormonal therapy to minimize side effects of medications that suppress estrogen (such as leuprolide acetate); add-back therapy usually decreases hot flashes and also helps prevent bone loss.
**11. Funding**	11.1 Describe the funding source(s) of the PVG and of the source guideline and their roles or any influences, in the PVG or guideline development processes, respectively. **Example** Supported by NCCN Foundation [15] The NCCN Foundation supports the mission of the National Comprehensive Cancer Network® (NCCN®) to improve the care of patients with cancer. One of its aims is to raise funds to create a library of books for patients. Learn more about the NCCN Foundation at NCCN.org/foundation. Funding: The consensus meeting in Zürich was financially supported by EULAR. There are no other financial disclosures. The sponsors had no role in voting, or in developing the final document [23].
**12. Conflicts of interest**	12.1 Report the conflicts of interests of contributors to the PVG and the source guideline in a format that the patients and the public can understand, and how they were managed. **Example** Competing interests: None [23].

### Rationale for the items in the RIGHT for PVG checklist

#### Basic information

We included five items in the basic information section. First, three items focus on information in the title or on the cover page that helps to identify the document as a PVG (items 1.1 to 1.3). An informative title that clearly states the topic and identifies the document as a PVG using an appropriate term can help readers locate and access the PVG effectively [24, 25], while the publication year and the version of a PVG help users know if they are using the most recent and valid edition [26]. Second, it is common for PVG users to seek additional information, thus contact information is helpful (item 2.1). Third, we suggest including a summary of the recommendations and other key points prior to the main text of the PVG (item 3.1). Such a summary can help the readers find the key information and promote the application of PVGs.

#### Background

A PVG may target a specific condition or a category, for example, prevention, diagnosis, or treatment [4]. PVGs should introduce the condition, the natural history, and the potential outcomes of the condition, as appropriate (item 4.1). PVGs that focus mainly on specific interventions for a condition, like behavioral interventions, should describe these interventions and how they might work (item 4.2).

PVGs are derivative products of the source guidelines (the guideline being translated); however, the scope and intended use are different from the source guidelines [3, 27]. Unlike clinical guidelines, the target populations in PVGs are mainly lay people including patients, their families, and the public [28, 29]. Some recommendations in the source guideline may not be relevant to a PVG. For example, a recommendation about how to prepare a biopsy would not be helpful, because patients would never discuss this with the pathologist [3]. Strictly professional practices which do not have any patient shared decision-making dimension do not need to be included in a PVG. We propose that a PVG should clarify the scope, purpose, and intended use together with the introduction to the topic (item 5.1). In addition, a reference or link to the source guideline is recommended (item 6.1), so that a reader can access the development process for the recommendations. A statement “this is the patient version of the [source guideline’s name] guideline” should be used. Any supplementary work that has contributed to the PVG in addition to the source guideline should be described accordingly [23].

#### Recommendations

The recommendations should indicate the specific target population and important outcomes, including both benefits, harms, and costs (if possible). If the recommendation is based on a comparison of two or more options in the source guideline, recommendations in the PVG should also report the alternative options (item 7.1). If undesirable outcomes are expected from the management options, the PVGs should describe any expected undesirable outcomes with potential management options based on what is mentioned in the source guideline (item 7.2). There is a clear need for PVGs to focus on self-management, as evidenced by results from the DECIDE (Developing and Evaluating Communication strategies to support Informed Decision and practice based on Evidence) project’s focus groups and user-testing [28] (item 7.3). We propose that PVGs should describe what actions patients or the public can take by themselves when this is reported in the source guideline, such as eating a balanced diet, reasonable exercising, and adopting other healthy lifestyle behavior. If a recommendation is going to give information on the effects or risks of interventions, numerical data appear to facilitate understanding by lay persons [30, 31]. Third, our project mainly focused what to report, while some other aspects, such as how to present numerical information, are not under the scope of this version. When deciding which recommendations to report in the PVG, the developers may consider focusing on those closely related to involvement by the patients or the public.

Each recommendation should be accompanied by the strength of recommendation and the assessment of the certainty (quality) of the evidence (item 13). Technical language such as that of the source guideline can be difficult for lay people to understand [27], however. Thus, a PVG should provide a clear and simple explanation of the meaning of any terms related to strength of the recommendations and certainty of the evidence, using visual aids if appropriate (see examples under item 8.1 in Table 1) [32].

Recommendations in PVGs should be easily identified by users. The developers should consider highlighting the recommendations with tables, boxes, bold type, or a distinctive color.

#### Other information

Other information proposed for reporting includes questions to ask clinicians (item 9.1), terms and abbreviations (item 10.1), funding source(s) and their roles for both PVG and the source guideline (item 11.1), and conflicts of interest of contributors to the PVG and the source guidelines (item 12.1). For the first point, a list of potential questions can encourage patient inquiries, allowing them to be more active during their healthcare visits and facilitating more effective communication and decision-making [33, 34]. For the second point, PVGs should avoid jargon and abbreviations when possible. Where unavoidable, the PVG should give clear definitions of new and key terms and explanations of abbreviations and acronyms. Additionally, a trustworthy guideline should report conflicts of interest transparently [35, 36], while the PVG should report the conflicts of interest in a format that patients and the public can easily understand and assess their potential impact. For example, if there were no conflicts of interest that might compromise public trust, the PVG should clearly report this [37]. If there are interests that were identified as conflicts in the source guideline that could impact the validity or credibility of the guideline, these should be clearly disclosed [38, 39] along with a brief explanation as to why the interest represents a conflict. For example, if the source guideline was funded by a company with a financial interest in one of the recommended interventions, the PVG should note this explicitly.

## Discussion

RIGHT-PVG is the first international consensus-based checklist for the reporting of PVGs. The checklist consists of 17 items under four domains. Application of the checklist aims to improve the transparent and understandable reporting of PVGs and facilitate their effective use among patients and the public. Use of the checklist could, in turn, promote shared decision-making and improved patient health outcomes [40]. Potential users of this checklist are the developers of PVGs, editors, and peer reviewers when assessing the reporting of PVGs, as well as researchers interested in PVGs. While the checklist does not prescribe a specific reporting style, it is important that each item is clearly presented. The specific style of the PVG will depend highly on the end users’ needs.

During the development process, we gathered initial items from a sample of PVGs, documents from organizations publishing PVGs, methodological publications on PVGs, and the literature on patients’ needs. This enabled us to start with a list of potential topics and considerations. With limited publications on the methodology or reporting of PVGs [12], the Delphi process was used to derive expert opinion-based criteria [13]. For several candidate items, the panelists could not reach consensus on whether to include the item (Additional file 1: Appendix 6). The PVG developers may need to consider those items when relevant to their specific condition. One example is whether to report the cost of each option. The debate mostly focused on the variability of costs of an intervention across jurisdictions and over time [41, 42].

There are several limitations to this work. First, we have not yet conducted any usability testing to understand how RIGHT-PVG works in practice. Second, we did not carry out face-to-face panel meetings due to a restricted budget, which might have limited the discussion among panelists. However, the two-round Delphi achieved a high level of consensus on the checklist and all panelists were in agreement with the final list of items, as were the external reviews. Third, our project mainly focused on what to report in the PVG; for issues about the style and presentation of the content, such as how to present numerical information, we suggest referring to the G-I-N public toolkit [3] and other relevant checklists [43]. Fourth, although the original checklist was informed by relevant studies about patients’ needs and preferences, and three representatives of the public participated in the Delphi panel, the majority of panelists were experts and it is possible that the views of lay people may have been under-represented. In the next phase, we plan to evaluate the validity, acceptability, and applicability of the checklist in different contexts [12]. We will collect feedback from PVG producers and the end users of PVGs (i.e., health professionals who discuss or disseminate PVG with patients, and patients and the public who use PVGs) for updating of this checklist in the future. Feedback from researchers using the RIGHT for PVG checklist and the continuously evolving evidence base on PVGs will also help with its refinement. We will upload all published documents on the RIGHT website page for RIGHT-PVG [44] and collaborate with colleagues who want to translate or adapt this tool for their specific contexts.

## Conclusion

The RIGHT for PVG reporting checklist provides an international consensus on important criteria when reporting PVGs. With this checklist, developers of PVGs can improve the transparency, consistency, and usability of their guidelines, which may ultimately lead to improved shared decision-making between patients and clinicians and improved patient health outcomes.

## Supplementary Information

**Additional file 1.**

## Data Availability

All data generated or analyzed during this study are included in this article and its supplementary information files.

## Abbreviations

RIGHT: The Reporting Tool for Practice Guidelines in Health Care
PVGs: Public or patient versions of guidelines
GRADE: Grading of Recommendations Assessment, Development and Evaluation
ACP: American College of Physicians
NICE: The National Institute for Health and Care Excellence
SIGN: The Scottish Intercollegiate Guidelines Network
AAN: The American Academy of Neurology
NCCN: The National Comprehensive Cancer Network
