# Supplementary material for: The reporting checklist for public versions of guidelines: RIGHT-PVG
Source: Implement Sci. 2021 Jan 11;16:10. doi: 10.1186/s13012-020-01066-z (PMC7798200; doi:10.1186/s13012-020-01066-z)
Supplement: Supplementary file 1 — Additional file 1. [file 13012_2020_1066_MOESM1_ESM.docx]

**Supporting information**

**Appendix 1 Basic information on the 30 sampled PVGs**

| Title | Developer | Publication year | Length (number of pages) | Topic | Format |
| --- | --- | --- | --- | --- | --- |
| 1. QEEG in ADHD Diagnosis – Summary of Practice Advisory for Patients and their Families (2016) | American Academy of Neurology (AAN) | 2016 | 2 | ADHD | Q&A |
| 1. Use of botulinum neurotoxin injections to treat movement disorders (2008) | AAN | 2008 | 2 | Movement disorders | Q&A |
| 1. Cervical Cancer Screening in Average-Risk Women | American College of Physicians (ACP) | 2015 | 1 | Cervical Cancer | Q&A |
| 1. Noninvasive Treatments for Acute, Subacute, and Chronic Low Back Pain | ACP | 2017 | 2 | Low Back Pain | Q&A |
| 1. Caring for the Patient with Cancer at Home: A Guide for Patients and Families (2015) | American Cancer Society (ACS) | 2015 | 90 | Cancer | Q&A |
| 1. Nutrition for the Person with Cancer During Treatment: A Guide for Patients and Families | ACS | 2015 | 45 | Cancer | Statement |
| 1. Managing Pancreatic Cysts: A Patient Guide (2015) | American Gastroenterological Association (AGA) | 2015 | 2 | Pancreatic cysts | Statement |
| 1. Managing Microscopic Colitis: A Patient Guide (2016) | AGA | 2016 | 1 | Colitis | Q&A |
| 1. A Patient Guide to Brain Stent Placement (2009) | University of Minnesota (authors’ address) | 2009 | 3 | Brain stent placement | Q&A |
| 1. Questions and answers on chemoprevention and breast cancer A guide for women and their physicians | Health Canada, Canadian Medical Association | 2001 | Long version: 47; Short version: 2 | Breast cancer | Q&A |
| 1. Parent Information: Breastfeeding your baby | Queensland Department of Health | 2016 | 2 | Breastfeeding | Q&A |
| 1. Patient information: Queensland Clinical Guidelines Hypoxic-ischemic encephalopathy (HIE) | Queensland Department of Health | 2016 | 2 | Hypoxic-ischemic encephalopathy (HIE) | Q&A |
| 1. Aspirin Use for the Primary Prevention of Cardiovascular Disease and Colorectal Cancer | The U.S. Preventive Services Task Force (USPSTF) | 2016 | 4 | Cardiovascular disease and colorectal cancer | Statement |
| 1. Screening for Depression in Adults | USPSTF | 2016 | 4 | Depression | Statement |
| 1. Information for you-Pregnancy and breast cancer (2014) | Royal College of Obstetricians & Gynaecologists (RCOG) | 2014 | 5 | Breast cancer | Q&A |
| 1. Oncoplastic breast reconstruction: guidelines for best practice – Information for patients (2012) | Breast cancer care  British Association of Plastic Reconstructive and Aesthetic Surgeons  Association of Breast surgery | 2012 | 8 | Oncoplastic breast reconstruction | Statement |
| 1. Breast Cancer – Trusted Information to Help Manage Your Care from the American Society of Clinical Oncology | The American Society of Clinical Oncology (ASCO) | 2017 | 55 | Breast Cancer | Q&A |
| 1. Incontinence Patient Guide | American Urological Association (AUA) | 2016 | 12 | Incontinence | Q&A |
| 1. Hypertension – Detection, Diagnosis and Management – A Guide for Patients(2008) | British Columbia Medical Association | 2008 | 4 | Hypertension | Q&A |
| 1. Depression in Children and Youth – A Guide for Parents (2009) | British Columbia Medical Association | 2009 | 1 | Depression | Statement |
| 1. Understanding Chemotherapy- A guide for people with cancer, their families and friends | Cancer Council Australia | 2014 | 60 | Chemotherapy | Q&A |
| 1. Information for women with endometriosis | The European Society of Human Reproduction and Embryology (ESHRE) | 2014 | 45 | Endometriosis | Q&A |
| 1. Prostate cancer: a guide for patients | European Society for Medical Oncology (ESMO) | 2016 | 32 | Prostate cancer | Q&A |
| 1. Kidney Cancer – NCCN Guidelines for Patients | National Comprehensive Cancer Network (NCCN) | 2015 | 86 | Kidney Cancer | Q&A |
| 1. Health services for people with sarcoma-Understanding NICE guidance – information for the public | National Institute for Health and Care Excellence (NICE) | 2006 | 4 | Sarcoma | Statement |
| 1. Glaucoma Referral and safe discharge-A booklet for patients, their families and carers | Scottish Intercollegiate Guidelines Network (SIGN) | 2015 | 28 | Glaucoma | Q&A |
| 1. The Hormone Foundation’s – Patient Guide to Androgen Deficiency Syndromes in Adult Men | The Hormone Foundation, The Endocrine Society | 2006 | 2 | Androgen deficiency syndromes | Q&A |
| 1. Diabetes and foot care: A patient’s checklist | Canadian Diabetes Association (CDA) | 2014 | 1 | Diabetes | Statement |
| 1. Patient Guidelines for the Prevention of Osteoporosis in Women | British Columbia Cancer Agency (BCCA) | 2011 | 2 | Osteoporosis | Statement |
| 1. Pregnancy Your Baby’s Movements and What They Mean | Australia and New Zealand Stillbirth Alliance (ANZSA) | 2012 | 2 | Baby’s movements during pregnancy | Q&A |

Q&A: questions and answers

**Appendix 2 Search strategy for studies on patients’ needs and studies relevant to the reporting and developing PVG**

| **Search strategy for studies on patients’ needs** **and studies relevant to the reporting and developing methods of PVG** |
| --- |
| #1 "patient perspective*"[Title/Abstract]) OR "patients perspective*"[Title/Abstract] OR "patients' perspective*"[Title/Abstract] OR "patient's perspective*"[Title/Abstract] OR "user perspective*"[Title/Abstract] OR "users perspective*"[Title/Abstract] OR "users' perspective*"[Title/Abstract] OR "user's perspective*"[Title/Abstract] OR "patient perce*"[Title/Abstract] OR "patients perce*"[Title/Abstract] OR "patients' perce*"[Title/Abstract] OR "patient's perce*"[Title/Abstract] OR "health perception*"[Title/Abstract] OR "user perce*"[Title/Abstract] OR "users perce*"[Title/Abstract] OR "users' perce*"[Title/Abstract] OR "user's perce*"[Title/Abstract] OR "user view*"[Title/Abstract] OR "users view*"[Title/Abstract] OR "users' view*"[Title/Abstract] OR "user's view*"[Title/Abstract] OR "patient view*"[Title/Abstract] OR "patients view*"[Title/Abstract] OR "patients' view*"[Title/Abstract] OR "patient's view*"[Title/Abstract] OR preference*[Title/Abstract] OR choice[Title] OR choices[Title] OR value*[Title] OR "health state values"[Title/Abstract] OR valuation*[Title] OR expectation*[Title/Abstract] OR attitude*[Title/Abstract] OR acceptab*[Title/Abstract] OR knowledge[Title/Abstract] OR "point of view"[Title/Abstract] OR “Patient Preference”[Mesh] OR “Patient Participation”[Mesh] OR "Attitude to Health"[Mesh]  #2 "discrete choice*"[Title/Abstract] OR "decision board*"[Title/Abstract] OR "decision analy*"[Title/Abstract] OR decision-support[Title/Abstract] OR "decision tool*"[Title/Abstract] OR "decision aid*"[Title/Abstract] OR "Decision Making"[Mesh] OR discrete-choice*[Title/Abstract] OR (decision*[Title] AND mak*[Title]) OR “decision mak*”[Title/Abstract] OR “decisions mak*”[Title/Abstract]  #3 patient*[Title/Abstract] OR user*[Title/Abstract]  #4 #2 AND #3  #5 "Guidelines as Topic"[Mesh] OR guid*[Title/Abstract]  #6 #1 AND #4 AND #5 |

**Appendix 3 The consensus panelist group**

| Name | Institution | Research interest |
| --- | --- | --- |
| 1. Amir Qaseem | American College of Physicians, USA | RIGHT, Clinical Practice Guideline Development |
| 1. Yngve Falck-Ytter | University Hospitals Case Medical Center, USA | RIGHT, GRADE, Gastroenterology |
| 1. Faruque Ahmed | Centers for Disease Control and Prevention, USA | RIGHT, Clinical Practice Guideline Development |
| 1. Madelin Siedler | American Gastroenterology Association, USA | Health communication (act as public representative) |
| 1. Ana Marušić | University of Split School of Medicine, Croatia EQUATOR Network, Croatia | RIGHT, Clinical Practice Guideline Development |
| 1. Susan L Norris | Oregon Health & Science University, USA | RIGHT, Practice Guideline Development and Review |
| 1. Elie Akl | American University of Beirut Medical Centre, Lebanon | RIGHT, Public health and health policy |
| 1. Edwin Chan Shih-Yen | Office of Clinical Sciences, Duke-NUS Graduate Medical School, Singapore | RIGHT, Clinical decision-making |
| 1. Claire Glenton | Cochrane Norway, Norwegian Institute of Public Health, Norway | Implementation research and systematic review |
| 1. Joey Kwong | National Center for Child Health and Development; Cochrane Gynaecological Cancer Review Group, China | Cochrane Systematic Review |
| 1. Sarah Louise Barber | WHO Kobe Centre, Japan | RIGHT, economic evaluation, quality of care assessments |
| 1. Akiko Okumura | Medical Information Network Distribution Service (MINDS) Guideline Centre, Japan | Practice guideline development |
| 1. MYEONG SOO Lee | Korea Institute of Oriental Medicine, Korea | Systematic reviews and clinical trials |
| 1. Suodi Zhai | Peking University Third Hospital, China | Pharmacy Department and Clinical Practice Guideline Development |
| 1. Hongcai Shang | Tianjin University of Traditional Chinese Medicine, China | RIGHT, Evidence-based Chinese Medicine and PVG Development |
| 1. Mingming Zhang | Chinese Cochrane Centre, China | Patient involvement and patient safety (act as public representative) |
| 1. Yuanyuan Zhang | Lanzhou University, China | Humanities & social sciences, education (act as public representative) |

**Appendix 4 Documents and summaries about PVG development or reporting**

| **No** | **Organization** | **Documents or summary from the organization on PVG development or reporting** |
| --- | --- | --- |
|  | The American Society of Clinical Oncology (ASCO) | “*Recommendations from ASCO’s Clinical Practice Guidelines are incorporated into relevant topic areas on Cancer.Net, ASCO’s patient information website. Not all guidelines are covered; preference is given to those that have a strong patient component where the recommendations would help a patient make decisions about or better understand their options for care. When included on Cancer.Net, recommendations are written for a lay audience and explain important medical terms in plain language and avoid excessive medical jargon*.”  ——From [guidelines@asco.org](mailto:guidelines@asco.org) |
|  | European Society for Medical Oncology (ESMO) | No formal guidance document.  “*ESMO is in the process of shifting production of the patient guides to self-publishing/managing process. Unfortunately, we don't have a standard guide document for development of such material, but we are open to sharing our experience and procedures we abide as we found that some of the standard methodologies simply don't apply to the audience we aim to reach.*  *There are different instructions available on the web on how to write healthcare material, but most of such documents are from US settings with advice to write the text at the fifth grade level. However, in our community, we have very strong patient advocates who don't want to see writing at this level and demand for the writing style at a significantly higher level. We analyzed the situation and understood that our patient material is not a substitution for the primary set of information given by oncology professionals, so we are fine that our material is at a higher level but it doesn't mean it's so complex so no one can understand it. We implement several measures to enhance readability in the non-medically educated community.*  *We produce the patient guides through several circles. We first create a dedicated editorial group composed of oncology professionals, mainly those involved in creating a parent document, the Clinical Practice Guideline. We also collaborate with oncology nurses from the European Oncology Nursing Society (EONS) and relevant patient/patient advocacy organizations in particular cancer type. So we organize phone conferences with all stakeholders together and discuss what kind of guide we are aiming for. However, we try to keep some consistency from title to title, although we know that in some diseases it's not possible to be absolutely consistent.*  ***Content to report in the guide***  *We start each guide with a summary of key information.*  *We show them in a diagram anatomy of the particular organ.*  *Then we describe what is … cancer.*  *We then show a diagram of how common is that cancer type.*  *Then we provide a set of information about what causes that cancer type.*  *The next section is devoted to how this cancer is diagnosed.*  *Then we provide a set of information on how the treatment will be determined.*  *Then we provide information about the treatment options.*  *The next section is about the possible side effects of treatment.*  *The next section is what happens when the treatment is finished.*  *The next chapter is about support groups.*  *We provide brief references that support the material development.*  *The final section is dedicated to the glossary. In the guide, we label difficult words with a particular color. In term of the glossary, you'll find different advice. In most cases, people say the writing should be simple, with explaining the difficult term in bracket immediately when it appears, but we found that such approach simply adds to the length of the booklet and not necessarily enhance readability.*  ***Presentation tips***  *“We select the supporting images for text and the images are subject of review by editorial board as well as the text. We seek opinions from our editorial group and combine their feedback and implement it to enhance readability. Some of our editorial groups run readability through differently available scores or simply ask the patients from their networks to pass through as well.*  *We absolutely appreciate a direct talk, using the phrases such as my treatment, my … instead of you or your.*  *We also use callouts from time to time to give visibility to important messages.*  *We try to arrange the final text with 20 lines of text per page. Many pages have less number of lines, we keep the space, as we have translations in many languages and some languages are lengthier than English.*”  *—*—From [clinicalguidelines@esmo.org](mailto:clinicalguidelines@esmo.org) |
|  | National Comprehensive Cancer Network (NCCN) | “*We do not have any such documentation available. The patient guidelines are based on the NCCN Clinical Practice Guidelines in Oncology, which are developed and updated by 54 individual panels comprising over 1,000 clinicians and oncology researchers from our 27 member institutions. Due to the extensive nature of this development process, we do not have any specific methodological documentation available for external use at this time.*”  *—*—From [patientguidelines@nccn.org](mailto:patientguidelines@nccn.org) |
|  | National Institute for Health and Care Excellence (NICE) | *Information for the public tab process notes* |
|  | Queensland clinical guidelines | *Parent information development guide* |
|  | Royal College of Obstetricians & Gynaecologists (RCOG) | <https://www.rcog.org.uk/en/patients/patient-leaflets/developing-patient-information/developing-patient-information-leaflets/> |
|  | Scottish Intercollegiate Guidelines Network (SIGN) | *SIGN 100: a handbook for patient and carer representatives* |
|  | Guidelines International Network and The European Society of Human Reproduction and Embryology (ESHRE) | *G-I-N Public Toolkit: Patient and Public Involvement in Guidelines 2015* |

**Appendix 5 Basic information of the 46 studies that contributed to initial items**

| No | Title | Journal | Author, year | Type of document | Topic |
| --- | --- | --- | --- | --- | --- |
| 1 | Standards for UNiversal reporting of patient Decision Aid Evaluation studies: the development of SUNDAE Checklist | BMJ Quality & Safety | Sepucha KR, 2017 | Reporting guideline | Decision aid |
| 2 | Development and validation of the guideline for reporting evidence-based practice educational interventions and teaching (GREET) | BMC Medical Education | Phillips AC, 2016 | Reporting guideline | Educational interventions |
| 3 | Balancing the presentation of information and options in patient decision aids: an updated review | BMC Medical Informatics & Decision Making | Abhyankar P, 2013 | Reporting guideline | Decision aid |
| 4 | A Reporting Tool for Practice Guidelines in Health Care: The RIGHT Statement | Ann Intern Med | Chen Y, 2017 | Reporting guideline | Practice guidelines |
| 5 | Improving the user experience of patient versions of clinical guidelines: user testing of a Scottish Intercollegiate Guideline Network (SIGN) patient version | BMC Health Services Research | Fearns N, 2015 | Relevant article for PVG development | PVG |
| 6 | Ensuring Quality Information for Patients: development and preliminary validation of a new instrument to improve the quality of written health care information | Health Expectations | Beki Moult BA, 2004 | Relevant article for PVG development | PVG |
| 7 | Patients, health information, and guidelines: A focus-group study | Scandinavian Journal of Primary Health Care | Liira H, 2015 | Relevant article for PVG development | PVG |
| 8 | How can clinical practice guidelines be adapted to facilitate shared decision making? A qualitative key-informant study | BMJ Quality & Safety | Van DWT, 2013 | Relevant article for PVG development | PVG |
| 9 | Public awareness about depression: the effectiveness of a patient guideline | International Journal of Psychiatry in Medicine | Patel VL, 2004 | Relevant article for PVG development | PVG |
| 10 | A systematic review of patient information leaflets for hypertension | Journal of Human Hypertension | Fitzmaurice DA, 2000 | Article on PVG methodology and reporting | PVG |
| 11 | Dissemination of Clinical Practice Guidelines: A Content Analysis of Patient Versions | Medical Decision Making an International Journal of the Society for Medical Decision Making | Santesso N, 2016 | Article on PVG methodology and reporting | PVG/public information |
| 12 | A colorectal cancer patient focus group develops an information package | Annals of the Royal College of Surgeons of England | Carney L, 2006 | Article on development of patient opinion-based information | PVG/public information |
| 13 | A qualitative study of Telehealth patient information leaflets (TILs): are we giving patients enough information? | BMC Health Services Research | Kayyali R, 2017 | Development of patient opinion based information | PVG/public information |
| 14 | Communicating cancer treatment information using the Web: utilizing the patient’s perspective in website development | BMC Medical Informatics and Decision Making | Hopmans W, 2014 | Patient opinion-based information development | PVG/public information |
| 15 | Information needs of cancer patients in west Scotland: cross sectional survey of patients' views | BMJ | Meredith C, 1996 | Patient opinion-based information development | PVG/public information |
| 16 | Information needs of people with low back pain for an online resource: a qualitative study of consumer views | Disability & Rehabilitation | Nielsen M, 2014 | Patient opinion-based information development | PVG/public information |
| 17 | ‘It made you think twice’ – an interview study of women’s perception of a web-based decision aid concerning screening and diagnostic testing for fetal anomalies | BMC Pregnancy & Childbirth | Annika Å, 2016 | Patient opinion-based information development | Decision aid |
| 18 | Use of a corporate needs assessment to define the information requirements of an arthritis resource centre in Birmingham: comparison of patients' and professionals' views | Rheumatology | Adab P, 2004 | Patient opinion-based information development | PVG/public information |
| 19 | Patient-controlled analgesia: what information does the patient want? | Journal of Advanced Nursing | Rgn GMC, 2002 | Patient opinion-based information development | PVG/public information |
| 20 | Ask the patients-they may want to know more than you think | BMJ | Dickinson D, 2003 | Patient opinion-based information development | Patient information need |
| 21 | Professional and patient perspectives on nutritional needs of patients with cancer | Oncology Nursing Forum | Hartmuller VW, 2004 | Patient opinion-based information development | PVG/public information |
| 22 | Patient and public attitudes to and awareness of clinical practice guidelines: a systematic review with thematic and narrative syntheses | BMC Health Services Research | Loudon K, 2014 | Patient opinion-based information development | PVG/public information |
| 23 | When Patients Write the Guidelines: Patient Panel Recommendations for the Treatment of Rheumatoid Arthritis | Arthritis Care & Research | Fraenkel L, 2016 | Patient opinion-based information development | PVG |
| 24 | Lay perceptions of evidence-based information--a qualitative evaluation of a website for back pain sufferers | BMC Health Services Research | Glenton C, 2006 | Patient opinion-based information development | PVG |
| 25 | Patients' need for information about cancer therapy | Oncology Nursing Forum | Skalla KA, 2004 | Patient opinion-based information development | Patient information need |
| 26 | Preventive health information on the Internet: Qualitative study of consumers' perspectives | Canadian Family Physician | Quintana Y, 2001 | Patient opinion-based information development | PVG/public information |
| 27 | Elaborating patient information with patients themselves: lessons from a cancer treatment focus group | Health Expectations | Moumjid N, 2003 | Patient opinion-based information development | PVG/public information |
| 28 | Development and evaluation of written patient information for endoscopic procedures | Endoscopy | Aabakken L, 2008 | Patient opinion-based information development | PVG/public information |
| 29 | What are cancer patients' experiences and preferences for the provision of written information in the palliative care setting? A focus group study | Palliative Medicine | Tomlinson K, 2012 | Patient opinion-based information development | PVG/public information |
| 30 | A randomised study of the impact of different styles of patient information leaflets for randomised controlled trials on children’s understanding | Archives of Disease in Childhood | Barnett K, 2005 | Patient opinion-based information development | PVG/public information |
| 31 | Suits you? A qualitative study exploring preferences regarding the tailoring of consumer medicines information | International Journal of Pharmacy Practice | Dickinson R, 2013 | Patient opinion-based information development | PVG/public information |
| 32 | Development of a series of patient information leaflets for constipation using a range of cognitive interview techniques: LIFELAX | BMC Health Services Research | Lake AA, 2007 | Patient opinion-based information development | PVG/public information |
| 33 | Patient information leaflets: informing or frightening? A focus group study exploring patients’ emotional reactions and subsequent behavior towards package leaflets of commonly prescribed medications in family practices | BMC Family Practice | Herber OR, 2014 | Patient opinion-based information development | PVG/public information |
| 34 | Words or numbers? Communicating risk of adverse effects in written consumer health information: a systematic review and meta-analysis | BMC Med Inform Decis Mak | Büchter RB, 2014 | Patient opinion-based information development | PVG/public information |
| 35 | Health Education Materials for Arab Patients: Content and Design Preferences | Medical Principles & Practice International Journal of the Kuwait University Health Science Centre | Hashim MJ, 2013 | Patient opinion-based information development | PVG/public information |
| 36 | Written information about individual medicines for consumers | Cochrane Database of Systematic Reviews | Nicolson D, 2009 | Patient opinion-based information | PVG/public information |
| 37 | A summary to communicate evidence from systematic reviews to the public improved understanding and accessibility of information: a randomized controlled trial | Journal of Clinical Epidemiology | Santesso N, 2015 | Patient opinion-based information | PVG/public information |
| 38 | What information is used in treatment decision aids? A systematic review of the types of evidence populating health decision aids | BMC Medical Informatics & Decision Making | Clifford AM, 2017 | Other methodological study | Decision aid |
| 39 | A systematic development process for patient decision aids | BMC Medical Informatics & Decision Making | Coulter A, 2013 | Other methodology paper | Decision aid |
| 40 | A ‘combined framework’ approach to developing a patient decision aid: the PANDAs model | BMC Health Services Research | Ng CJ, 2014 | Other methodology paper | Decision aid |
| 41 | Quality of reporting of patient decision aids in recent randomized controlled trials: A descriptive synthesis and comparative analysis | Patient Education & Counseling | Lewis KB, 2017 | Other methodology paper | Decision aid |
| 42 | Communicating evidence for participatory decision making | JAMA | Epstein RM, 2004 | Other methodology paper | patient information need |
| 43 | A systematic review on communicating with patients about evidence | Journal of Evaluation in Clinical Practice | Trevena LJ, 2006 | Other methodology paper | patient information need |
| 44 | Informing patients: the influence of numeracy, framing, and format of side effect information on risk perceptions | Medical Decision Making an International Journal of the Society for Medical Decision Making | Peters E, 2011 | Other methodology paper | patient information need |
| 45 | An empirical study of patient participation in guideline development: exploring the potential for articulating patient knowledge in evidence-based epistemic settings | Health Expectations | Hm VDB, 2013 | Other methodology paper | patient participation in guideline development |
| 46 | Reproductive Decision Support: Preferences and Needs of Couples at Risk for Hereditary Cancer and Clinical Geneticists | Journal of Genetic Counseling | Reumkens K, 2018 | Other methodology paper | Decision aid |

**Appendix 6 Items for which the panelist did not reach consensus**

| **Section/Topic** | **Items** |
| --- | --- |
| ***Basic information*** | |
| **Report the information of the institution and contributors** | 1. Report the institution or organization that developed the PVG, and describe participation of stakeholders in the development of PVG, including their specialties and their role in the development process. |
| **Summary** | 1. Provide a table of contents of the PVG when needed, i.e. when the PVG is longer than two pages and locating the information of interest without a table of contents would be difficult |
| ***Background*** | |
| **Users and setting** | 1. Describe the group of primary users of the PVG, e.g. patients and their families, or general public. |
|  | 1. Describe the target setting of the PVG, e.g. remote areas. |
| **Behavior and lifestyle related to the condition** | 1. Describe the contributory lifestyle factors, such as a balanced diet, reasonable exercise, and healthy behavior, for the conditions from the source guideline when applicable. |
| ***Methods*** | |
| **How are the PVG produced?** | 1. Indicate how the recommendations and outcomes in the PVG were selected. |
| **Update plan** | 1. Describe the update plan of the PVG, including when and how it will be updated. |
| **Quality assurance** | 1. Describe the reviewers and the review process of the PVG (possibly as an appendix or a link that the users can easily access). |
| ***Recommendations*** | |
| **Highlight recommendations where applicable** | 1. Describe the potential predictors that may affect patient outcomes, such as the characteristics of the patient, family and carers. |
| **Cost-effectiveness and cost** | 1. Describe the approximate cost-effectiveness of each option if the source guideline provided sufficient information. One may also consider reporting the cost when applicable (and other details such as whether it is covered by insurance). |
| **Preferences and values** | 1. Describe the process of considering patients’ preferences and values (P&V), including the method used to collect P&V information and content of P&V. |
| **Availability and accessibility** | 1. Describe the local availability of the management or intervention options (e.g. treatment) if possible. |
| **Feasibility** | 1. Describe the information about feasibility related to the recommended options when applicable. |
| ***Other Information*** | |
| **Reference list** | 1. Provide a list of references documenting the underlying evidence. This can be included in the PVG document or provided as a link to the reference list. |
| **Sources of additional information** | 1. Provide links or other sources for users to obtain more information or get more help. For example, relevant sources in the original clinical practice guideline. |
